# Development of a Noninvasive Genotyping‐In‐Thousands (GTseq) Panel for Long‐Term Conservation of Western Great Lakes Gray Wolves (*Canis lupus*)

**DOI:** 10.1002/ece3.71240

**Published:** 2025-04-21

**Authors:** Samuel D. Hervey, Bridgett M. vonHoldt, Mark C. Romanski, Tyler J. Wheeldon, Brent R. Patterson, Kristin E. Brzeski

**Affiliations:** ^1^ College of Forest Resources and Environmental Sciences Michigan Technological University Houghton Michigan USA; ^2^ Department of Ecology and Evolutionary Biology Princeton University Princeton New Jersey USA; ^3^ Isle Royale National Park National Park Service Houghton Michigan USA; ^4^ Wildlife Research and Monitoring Section Ontario Ministry of Natural Resources Peterborough Ontario USA

**Keywords:** *Canis*, GTseq, microhaplotype, monitoring, noninvasive, SNP

## Abstract

The application of noninvasive genetic methods toward the field of conservation has increased our understanding of many wildlife populations that are difficult to sample, allowing for better management. In molecular ecology, the use of noninvasive sampling became widely feasible with the advent of microsatellites, a highly polymorphic, short‐length marker that could be genotyped from low‐quality DNA sources. Despite decades of use, many microsatellite panels continue to suffer from high genotyping error rates, allelic dropout, and limited reproducibility across laboratories. To address these issues, single nucleotide polymorphisms (SNPs) offer advantages such as lower genotyping error rates, avoidance of allelic dropout due to consistent allele length, and automated calling through bioinformatic pipelines, reducing human subjectivity and error. Given the advantages SNPs provide relative to microsatellites as a molecular marker, the use of SNP panels and specifically, the method of genotyping‐in‐thousands by sequencing (GTseq) has gained popularity. Here, we developed a GTseq panel for western Great Lakes canids comprised of 196 loci, capable of species identification, accurately inferring sex (97.2%), identifying unique individuals (probability of identity = 6.71e^−41^), assigning relationships (false positive rate = 9.34e^−14^), and assigning genotypes with low error (0.39%). In an attempt to improve genotyping success with low‐quality samples, we found that while increasing the number of PCR cycles yielded a higher percentage of genotyped loci, it also increased on‐target reads in negative PCR controls. We suggest approaching this manipulation with caution and emphasize the importance of including and reporting negative PCR controls. Further, quantitative PCR was a powerful method to estimate host‐specific DNA concentrations, enabling conservative sample selection for library preparation with respect to GTseq affordability.

## Graphical abstract

This study presents a novel genotyping‐in‐thousands by sequencing (GTseq) method for noninvasive genetic research on western Great Lakes (WGL) canids, addressing key challenges with low‐quality DNA samples. The WGL GTseq panel can differentiate multiple canid species (i.e., gray wolf, eastern wolf, coyote, dog, and red fox) and infer sex while having greater resolution to identify unique individuals and assign relationships when compared to microsatellites. The WGL GTseq panel aligns with the evolving field of noninvasive genetics, offering a standardized approach to produce accurate and reproducible genotypes (genotyping error rate = 0.39%) essential for long‐term noninvasive monitoring programs.

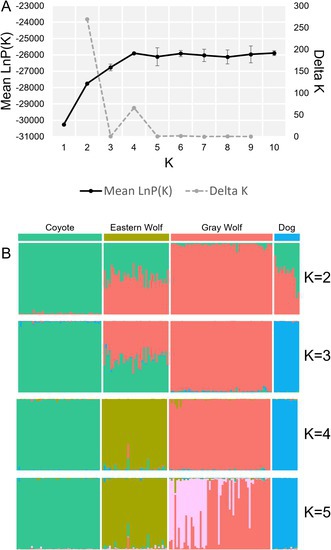

## 1 Introduction

Noninvasive genetic sampling has fundamentally transformed the study and management of free‐ranging populations, becoming a ubiquitous tool among researchers and managers alike for understanding and conserving wildlife. The application of noninvasive genetics has the advantage of both reducing wildlife stress during sampling while enhancing and refining the questions that can be answered from a single sample such as individual reidentification (e.g., Shyvers et al. 2019), parentage (e.g., Adams et al. 2011), ancestry (e.g., Barnes et al. 2022), and diet (e.g., Shi et al. 2021).

The widespread application of noninvasive genetic tools in conservation can largely be attributed to the historic focus on microsatellites, which are short repeat sequences (e.g., di‐, tri‐, or tetranucleotide repeats) of DNA (Wyman and White 1980). Microsatellites are highly polymorphic, short fragments (approximately 100–300 bp) amplified through targeted polymerase chain reaction (PCR), which made them well suited for poor quality noninvasive sources of low copy, sheared, or degraded DNA, such as scat (e.g., Adams et al. 2011), hair (e.g., Lorenzini et al. 2004), shed skin (Villarreal et al. 1996), feathers (e.g., Hovarth et al. 2005), hatch membranes (e.g., Hervey et al. 2019), and eggshells (e.g., Egloff et al. 2009). The development and application of microsatellites for noninvasive genetic research have unquestionably advanced our understanding of many species globally and have been a critical tool to help manage some of the most endangered species suffering from low genetic diversity and high inbreeding (e.g., Koike et al. 2008; Brzeski et al. 2014; Zhang et al. 2019). Yet microsatellites have some significant drawbacks, such as genotyping errors, allelic dropout, and homoplasy, but perhaps most limiting is the effort required to confidently compare genotyping results between laboratories when using capillary electrophoresis (Pasqualotto et al. 2007; Fabbri et al. 2012).

Single nucleotide polymorphisms (SNPs) are an alternative molecular marker that has rapidly gained popularity in noninvasive work (e.g., Eriksson et al. 2020; Schmidt et al. 2020; Harmoinen et al. 2021; Hayward et al. 2022). SNPs lack the limitations common to microsatellites, including a higher genotyping success rate when using low‐quality DNA (Campbell and Narum 2009), no allelic dropout due to varying lengths of alleles, and genotypes that are called using a bioinformatic pipeline, which removes human subjectivity and error. SNPs are also found throughout the genome, allowing researchers to explore similar questions microsatellites once provided from neutral marker sets, while also being capable of monitoring traits of interest similar to forensic DNA phenotyping (Tozzo et al. 2021). Also, when limited to a few hundred loci, SNP panels can be designed to be amplified in a single PCR multiplex reaction and sequenced using high‐throughput sequencing platforms, which has allowed researchers to substantially reduce the cost per sample while maintaining the flexibility to select SNPs that are most informative toward their research questions (Campbell et al. 2015; Meek and Larson 2019).

The method of amplifying hundreds of SNPs in a single PCR reaction, referred to as genotyping‐in‐thousands by sequencing (GTseq), has found increased use in many research programs ranging from stock identification in walleye ( *Sander vitreus* ; Bootsma et al. 2020) to identity analysis for gray wolves ( *Canis lupus* ) to associate with variation in diet (Roffler et al. 2023). In addition to the wide range of applications GTseq holds for the field of conservation, its ability to have data generated in a reproducible fashion allows laboratories spanning large geographic regions and political boundaries to merge datasets, providing a more comprehensive understanding of the species or population of interest (Euclide et al. 2022).

The field is currently at a tipping point in method development for noninvasive genetic studies, where the advantage that SNPs present relative to microsatellites is moving the field from microsatellite to SNP‐based panels. While GTseq and other SNP‐based assays hold great promise for noninvasive genetics, there remain challenges associated with optimizing these methods for low‐quality or degraded DNA samples, as well as the need to validate the results and inference from new SNP panels.

Here, we describe the steps and considerations when designing a GTseq panel for noninvasive samples, to study gray wolves of the western Great Lakes (WGL) with respect to our goals of species identification, sex, identity, and kinship. Gray wolves of the WGL have a unique mosaic of ancestry containing admixture from both eastern wolves ( *C. lycaon* ) and coyotes ( *C. latrans* ; Heppenheimer et al. 2018; von Holdt et al. 2016). Given the varying levels of conservation concern among these taxa, it is imperative that accurate species identification is achieved through this GTseq panel. Further, the GTseq panel design incorporated the use of microhaplotypes (multiple unlinked proximal SNPs; Kidd et al. 2013) to reduce the rate of fixation of alleles when studying small populations likely to become inbred through time such as the Isle Royale gray wolf population (Hervey et al. 2021).

We also outline the challenges associated with GTseq panel optimization when using DNA extracted from low‐quality sources. Specifically, we discuss the benefit of pre‐screening DNA for samples unlikely to amplify and how manipulation of GTseq library preparation steps can lead to a higher percentage of on‐target reads and genotyping success. We demonstrate the WGL GTseq panel is comparable to other common genetic methods with the added benefits that it can be optimized for high and low‐quality samples, is high throughput, and generates genotypes in a reproducible fashion. Our research is at the forefront of a growing effort to implement novel and important tools in the future of noninvasive molecular ecology and wildlife management.

## 2 Materials and Methods

### 2.1 Overview of GTseq Panel Development and Considerations

**FIGURE 1:**
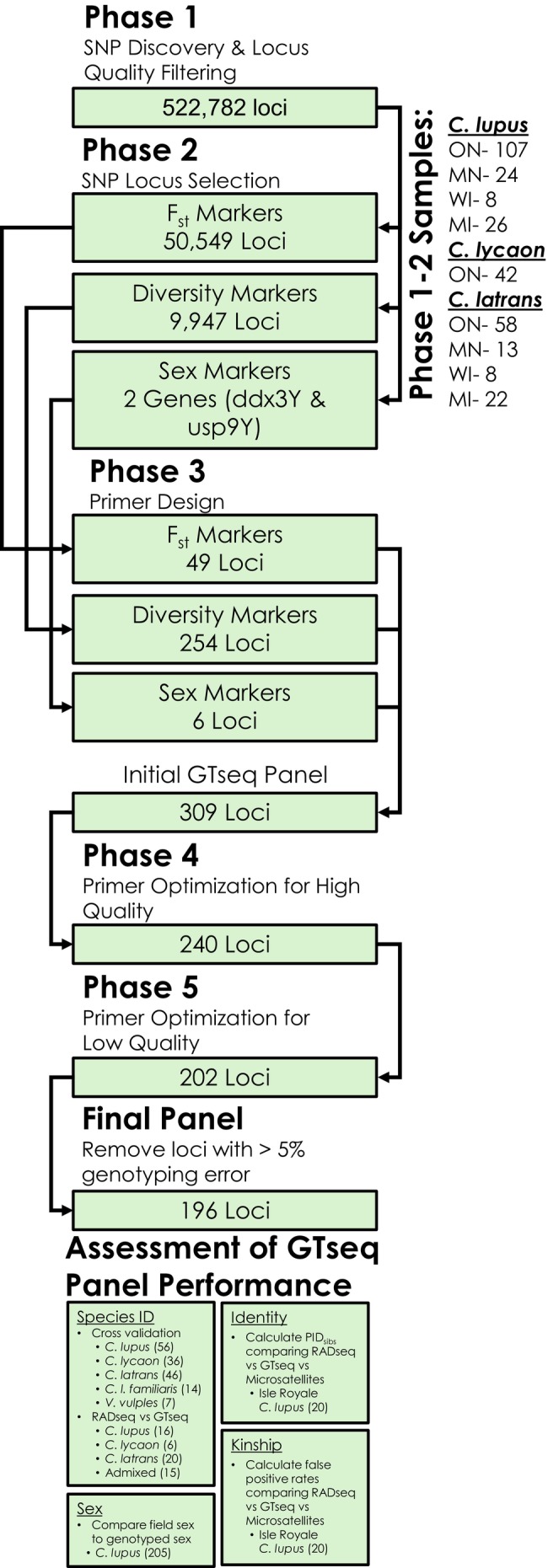
Analytical workflow for locus discovery, selection, filtering, and assessment for the western Great Lakes gray wolf GTseq panel. The number of canid samples used for each phase or analysis is listed with sample sizes listed in parentheses.

We implemented five phases to select loci that can differentiate canids, infer sex, identify unique individuals, and assign kinship in the final GTseq panel: (1) *Candidate SNP discovery and locus quality filtering*, (*2*) *SNP locus selection*, (3) *primer design*, (4) *primer optimization for high‐quality samples*, and (5) *primer optimization and methodological considerations for low‐quality samples* (Figure 1). These objectives required a detailed assessment of SNP allelic variation within and among populations, with a subset specifically to be physically located on the Y chromosome for sex inference. We further made use of several preexisting, publicly available datasets that were generated using a reduced representation approach, specifically restriction site‐associated DNA sequencing (RADseq).

#### 2.1.1 Phase 1: Candidate SNP Discovery and Locus Quality Filtering

We generated a list of candidate SNP loci from analyzing RADseq genotypes for canids from the WGL. First, we extracted high molecular weight genomic DNA derived from blood or tissue using a DNeasy Blood and Tissue Kit (Qiagen, Maryland, USA) or the BioSprint 96 DNA Blood Kit (Qiagen) in conjunction with a KingFisher Flex Purification platform (Thermo Fisher Scientific, Waltham, MA, USA) following manufacturers' protocols from 308 wild canids representing the *Canis* species diversity in the western Great Lakes: 165 gray wolves (Ontario, *n* = 107; Minnesota, *n* = 24; Wisconsin, *n* = 8; the Upper Peninsula [UP] of Michigan, *n* = 26), 101 coyotes (Ontario, *n* = 58; Minnesota, *n* = 13; Wisconsin, *n* = 8; Michigan, *n* = 22), and 42 eastern wolves from Ontario. Samples were obtained from state management programs as well as government and state organizations (e.g., US Department of Agriculture, Department of Natural Resources, Ontario Ministry of Natural Resources, and furbearer programs). In all cases, the origin of each sample was documented at the state or provincial level and linked either to a specific GPS location from the exact collection site or to the nearest township.

We assessed DNA concentration using either PicoGreen or Qubit 3.0 fluorometry systems (Thermo Fisher Scientific, Waltham, MA, USA). We prepared genomic libraries following a modified RADseq approach (Ali et al. 2016). Briefly, we first digested DNA with the *Sbf1* restriction enzyme, followed by ligating an 8 bp biotinylated, uniquely barcoded adapter to each sample. We equimolarly pooled up to 48 samples and subsequently sheared to 400 bp via sonication on a Covaris LE220 (PerkinElmer, Shelton, CT, USA) or Diagenode Bioruptor Pico (Hologic, Denville, NJ, USA). We enriched pools for the uniquely ligated adapters using a streptavidin bead‐binding assay (Dynabeads M‐280; Invitrogen, Thermo Fisher Scientific, Waltham, MA, USA) followed by using the NEBNext Ultra DNA Library Prep Kit (New England Biolabs, Ipswich, MA, USA). We used Agencourt AMPure XP beads (Beckman Coulter, Indianapolis, IN, USA) for all library purifications and for the size selection step for 2 × 150nt sequencing on an Illumina HiSeq 2500 or NovaSeq 6000 platform (Illumina, San Diego, CA, USA).

We retained only raw reads that contained an *Sbf1* restriction enzyme cut site and demultiplexed each pool using a custom perl script (flip_trim_sbfI_150821.pl.; https://doi.org/10.5281/zenodo.4673716). Next, we demultiplexed individual barcodes using the STACKS v2.3 (Rochette et al. 2018) *process_radtags* function with the following parameters: allowed no more than two mismatches, removed reads with uncalled bases, and excluded nucleotides with a quality score < 10. We then removed PCR duplicates with the *clone_filter* function using default parameters. Samples were mapped to the CanFam3.1 (GCA_000002285.2) reference genome (Lindblad‐Toh et al. 2005) and the annotated reference Y chromosome (KP081776.1; Li et al. 2013) using BWA‐MEM v0.7.12 (Li and Durbin 2009; Li 2013). We excluded reads with low‐quality mapping scores (MP < 20) using Samtools v1.9 (Li et al. 2009). After converting SAM to sorted BAM files, we first created a complete catalog of discovered SNP variants with the STACKS v2.3 *gstacks* function (using the default parameters of var‐alpha 0.05 and gt‐alpha 0.05) and inferred genotypes using the *populations* functions.

#### 2.1.2 Phase 2: SNP Locus Selection

We first excluded SNP loci that had a low minor allele frequency (MAF) less than 0.01 (–maf 0.01) or with more than 10% missingness across all samples (–geno 0.1) with PLINK v1.9 (Chang et al. 2015). We next generated a neutral set of SNPs by removing loci in linkage disequilibrium (LD) which was inferred by a high correlation coefficient (*r* ^*2*^ > 0.5) in a window‐based assessment (–indep‐pairwise 50 5 0.5) and loci that significantly deviated from Hardy–Weinberg equilibrium (–hwe 0.05). We used the R package *vcfR* (Knaus and Grunwald 2017) and removed loci mapped to multiple regions by visualizing the frequency of read depths and only retaining loci within the first of two distinct peaks, where the second peak contains loci having twice the sequencing fold coverage as those in the first peak and thus inferred to be due to non‐uniquely mapping reads.

Our first objective was to select loci capable of discerning the canid species. To ensure the reference canid population did not contain samples incorrectly assigned to a species group before SNP selection, we used a maximum likelihood clustering approach (ADMIXTURE v1.3.0; Alexander et al. 2009) to identify samples not adhering to their predefined species assignment. We assessed genetic cluster membership probability (*Q*) at three clusters (*K* = 3), assuming reference canids would have the highest probability of assignment to their respective cluster (reference species: coyotes, eastern wolves, and gray wolves). We excluded samples as possible reference canids that had a *Q* value higher in a cluster not matching their original species assignment. After establishing the canids in each reference group, we estimated pairwise population differentiation (*F*_ST_) with the R package *Adegenet* (Jombart 2008) and ranked the loci by decreasing *F*_*ST*_. We conducted three independent pairwise *F*_*ST*_ calculations: (1) all reference canids, (2) eastern and gray wolves, and (3) WGL gray wolves by location relative to Lake Superior (northeastern, northwestern, western, or southern Superior). We again ranked loci by decreasing *F*_*ST*_ and selected the top 30 loci from each grouping for a total of 90 loci for primer design.

Our next objective was to identify loci that would provide the necessary power for identity and kinship analyses within gray wolves of the WGL. Hence, we constructed a new subset of loci from the raw genotyping catalog by using the *populations* module in STACKS v2.3 and retained only gray wolves. We repeated the filtering for MAF and missingness but restricted our analyses to include only autosomal chromosomes (–chr 1–38). We utilized the service GTseek LLC (Twin Falls, ID, USA) for locus discovery, prioritizing loci based on diversity metrics (i.e., MAF, heterozygosity, allelic richness). We selected loci that formed a microhaplotype (a minimum of two SNPs within 100 nucleotides of each other and not in phase that can be captured with a single pair of primers). We first generated an allele frequency output file using VCFtools (Danecek et al. 2011) and then used a custom perl script to identify clusters of SNPs that would serve as microhaplotype markers based on the following parameters: each 100 base pair window must contain at least 1 SNP with a MAF between 0.2 and 0.45 and the standard deviation between the allele frequencies in the SNP cluster must be higher than 0.01 to avoid SNPs in perfect phase but not exclude too many candidates. We also supplemented the SNP selection with additional loci of high MAF (0.30–0.45) that were spaced at least 250Kb apart in the genome assembly (herein referred to as diversity markers).

For our last objective, we selected loci for inferring sex. We focused on scaffold sequences that have been documented to contain large fragments with only male sequence alignments. To do this, we masked the genome with RepeatMasker version 4.1.4 (https://www.repeatmasker.org/) using the flag –species canid and then targeted regions of the scaffold that contain male‐specific single copy genes (Smeds et al. 2019) and reconfirmed by comparing against the Y‐chromosome assembly for gray wolves (GCA_905319855.2). We further verified that these target sequences for sex markers did not also align to any of the autosomes. We subsequently designed primers for all markers that passed this method.

#### 2.1.3 Phase 3: Primer Design

We collaborated with GTseek to design primers that flank the candidate 303 autosomal markers (254 diversity and 49 *F*_*ST*_ markers) and six sex markers (see Results). GTseek uses a proprietary pipeline for multiplex PCR primer design. In brief, target‐specific primers were designed to be between 18 and 24 bases in size and have melt temperatures between 59°C and 60°C with GC content between 30% and 70%. All flanking primer sequences that met these criteria were then passed through a filtering script that checked each primer with its tag sequence for stable hairpin structures and 3′ binding sites on other primers. Primers were not considered for inclusion in the panel if the last 10 bases of their primer sequences had a binding site on any other primer with a melt temperature higher than 15°C or if any single primer with its tag sequence formed a stable hairpin structure with a melt temperature above 50°C. If a primer set passed all filtering criteria, then it was passed onto the full primer set for the panel. Possible off‐target amplification within the wolf genome or other prey species genomes was not considered as part of primer design.

The first set of primers included a target specific primer sequence and a single stranded overhang sequence to anneal to second round primers. The second set included indexing primers designed to contain the complement sequence to the single stranded overhang of amplicons from first round PCR to allow for binding while also containing a unique 6‐mer sequence for indexing samples followed by the P5 or P7 capture sequence for compatibility with Illumina sequencing platforms. This design effort produced 15 unique i7 indexing primers and 96 unique i5 primers that allow up to 1440 samples to be pooled in a single sequencing run (https://doi.org/10.5281/zenodo.4673716).

#### 2.1.4 Phase 4: Primer Optimization for High‐Quality Samples

We tested the initial 309 loci on 93 high‐quality (i.e., blood and tissue) canid DNA samples (> 5 ng/μL; 58 gray wolves, 18 coyotes, 5 eastern wolves, 11 admixed canids, and 1 red fox). We extracted DNA from each sample following the protocol described above. We used a two‐round PCR setup following the protocol of Campbell et al. (2015) with slight modification. Each sample was initially amplified in a single multiplex PCR reaction containing all 309 primer pairs (first round PCR or PCR1). Each well contained 3.5 μL of 2× Qiagen Multiplex PCR Plus Master Mix, 1.5 μL primer master mix (0.25 μM per primer), and 2.0 μL of template DNA. Every PCR plate prepared during PCR1 also contained a negative PCR control. We used the thermal cycler conditions of an initial denaturation of 95°C for 15 min followed by 5 cycles of [95°C for 30 s, 57°C for 30 s, and 72°C for 2 min]. We structured for the annealing temperature to have a slow cooling ramp rate of 5% (~0.1°–0.3° per second), followed by 13 cycles of [95°C for 30 s, 65°C for 30 s, and 72°C for 30 s].

Following PCR1, each sample was diluted 20‐fold before use as a template for the second round PCR (or PCR2), which consisted of annealing forward and reverse indexing primers to single‐stranded tails from PCR1 amplicons. Here, each well received 5 μL of 2× Qiagen Multiplex PCR Plus Master Mix, 1 μL (10 μM) of a forward indexing primer, 1 μL (10 μM) of a reverse indexing primer, and 3 μL of diluted PCR1 product. We used thermal cycler conditions of initial denaturation at 95°C for 15 min followed by 10 cycles of [95°C for 10 s, 65°C for 30 s, and 72°C for 30 s], with a final extension of 72°C for 5 min.

We cleaned and normalized PCR2 products using Just‐A‐Plate 96 PCR Purification and Normalization following the manufacturer's protocol (Charm Biotech, San Diego, CA, USA). We pooled 5 μL of clean PCR2 product from each well into a single tube. The single pooled library was quantified using Qubit 3.0 fluorometry (Thermo Fisher Scientific, Waltham, MA, USA) and loaded onto a MiSeq reagent cartridge v2 2 × 150nt with a 5% PhiX spike and a final loading concentration of 8 pM. Handling of raw FASTQ files was processed consistently among runs and described in the section *Bioinformatics for annotating and error checking genotypes*.

For the initial optimization, we worked with GTseek to remove any loci that were overrepresented in the sequencing data (indicating multiple genomic copies) and any locus primers that formed primer dimers that accounted for at least 1% of the raw sequencing reads. The GTseq panel retained 240 loci following the optimization run from high‐quality DNA (see Results).

#### 2.1.5 Phase 5: Primer Optimization and Methodological Considerations for Low‐Quality Samples

We tested a reduced set of 240 loci identified in Phase 4 on 556 canid scat samples collected from Isle Royale National Park and Voyageurs National Park across winter and summer months. Scat samples were either stored in Longmire buffer, desiccated, or frozen before processing. DNA extractions were performed in a separate laboratory space dedicated to extracting low copy DNA using a modified protocol from the DNeasy Blood and Tissue kit. Every batch of extractions (i.e., 23 samples) included a negative DNA extraction control. The absence of contamination was verified using qPCR by confirming the negative DNA extraction control showed no detectable host DNA concentration (see below for details on qPCR methods). We homogenized 200 mg of scat with 50 μL proteinase K, 500 μL buffer ATL, and 10–20 1.0 mm Zirconia/Silica beads via vortexing for 10 min followed by incubation at 56°C overnight. Additionally, we used 500 μL of buffer AL and 500 μL of 96% molecular‐grade ethanol. All remaining steps of the protocol followed the DNeasy Blood and Tissue kit protocol. All post‐PCR processing was completed in a separate room for PCR product handling, bead cleans, and normalization to avoid PCR or high copy DNA contamination.

We prepared all samples following the same library construction protocol for high‐quality DNA with an additional bead clean between PCR1 and PCR2 to increase the percentage of on‐target reads (reads aligned to the region of interest) by removing primer heterodimers after PCR1 (Eriksson et al. 2020). Specifically, we carried out bead cleans on 96‐well PCR plates by adding 1.4 volumes of Sera‐Mag SpeedBeads Carboxyl/hydrophobic beads (Thermo Fisher Scientific, Waltham, MA, USA) to the PCR1 product and washed with 70% molecular grade ethanol. We eluted the DNA from the beads in 20 μL of molecular‐grade water. We prepared these purified samples as described for high‐quality DNA. The final pooled library was quantified and sequenced using a NextSeq 2000 P2 2 × 150nt kit (Illumina) with a 1% phiX spike and a final loading concentration of 800 pM with onboard dilution and denaturation. We also loaded 600 μL of a 0.6 μM custom primer diluted in HT1 (hybridization buffer) onto the sequencing cartridge to allow the i5 index to be captured (https://doi.org/10.5281/zenodo.4673716).

The final stage of primer optimization with low‐quality DNA included a second iteration of aggressively removing loci with off‐target amplification. Specifically, we removed any locus with less than 10% on‐target amplification when using low‐quality samples, retaining 202 loci on the panel. In parallel with primer optimization, we tested four different methods, including sample preparation, number of loci amplified per PCR reaction, number of PCR1 cycles used, and DNA screening (Table 1).

**TABLE 1:** Reasoning and outcomes of manipulations made to increase the quality of libraries submitted for GTseq.

Manipulation	Reasoning	Qualitative outcome
Treat DNA with ExoSAP	Reduce partial amplification of highly sheared/fragmented DNA	ExoSap DNA treatment did not increase % on‐target reads
Split primer master mix	Reduce probability of primer dimers forming	Splitting MM did not increase % on‐target reads
Increase PCR1 Cycles	Increase % genotyped from low DNA concentrations	Increase in % genotyped and incorrectly assigned reads
Filter samples using qPCR	Remove samples not likely to contain host DNA	qPCR filtered samples increased overall library quality

First, given scat‐derived DNA is typically sheared with single‐end overhangs, we attempted to remove overhangs by treating 12 DNA samples with a homemade ExoSAP mix containing exonuclease I and shrimp alkaline phosphatase (New England BioLabs, Ipswich, MA, USA). We treated each DNA sample in four replicates, where we compared the percentage of on‐target reads for each sample with and without ExoSAP treatment. We calculated the change in on‐target reads by subtracting the percent on‐target reads from samples treated with ExoSap from the percent on‐target reads with no treatment. We used a one‐sample t‐test to test for significant differences.

Second, we split our PCR1 primer mix into two separate reactions to test if decreasing the number of primer sets would increase the percentage of on‐target reads. To examine the relationship between the number of primer mixes and the percentage of loci genotyped, while controlling for DNA concentration across samples, we strategically selected seven samples with DNA concentrations ranging from 0.007 to 59.190 ng/μL. Each sample was prepared using two different primer multiplex treatment groups: Treatment one (MM1) contained all primer pairs in a single PCR reaction, and Treatment two (MM2) received a random subset of half the primer pairs. For MM2, equal volumes of PCR1 product from each PCR reaction were pooled first before carrying out the bead clean step. We used a two‐sample t‐test to test for significant differences in the percentage of on‐target reads between MM1 and MM2.

Third, we prepared two PCR plates containing the same samples, where the first plate was prepared using the same library preparation steps described for low‐quality DNA where 13 PCR1 cycles were used, while the second plate increased the number of PCR1 cycles to 16. We used a Wilcoxon signed‐rank test where we specifically tested if 13 PCR1 cycles resulted in fewer loci genotyped relative to 16 cycles. We also observed the number of on‐target reads and percentage of on‐target reads for the negative PCR controls when using 13 and 16 cycles.

Finally, we tested if pre‐screening and measuring the concentration of host DNA allowed us to define minimum thresholds for successful genotyping. We used quantitative PCR (qPCR) to measure host DNA extracted from scat (Hayward et al. 2020). We selected a single locus from the GTseq panel that showed consistent amplification during optimization when using high‐quality DNA (chr16_54754713). Further, we specifically tested the locus to ensure it would amplify *Canis* DNA (https://doi.org/10.5281/zenodo.4673716). We created DNA standards using 20 ng/μL of DNA extracted from gray wolf tissue with four 10‐fold dilutions of the extract to have a total of five standards from 20 to 0.002 ng/μL. We prepared a qPCR master mix of 5 μL ZymoTaq qPCR Premix (2×), 0.33 μL chr16_54754713 forward primer (10 μM), 0.33 μL chr16_54754713 reverse primer (10 μM), 3.34 μL molecular‐grade water, and 1 μL DNA template per reaction. On a StepOne real‐time PCR system (Thermo Fisher Scientific, Waltham, MA, USA), we used the cycler conditions of 95°C for 10 min, 40 cycles of [95°C for 30 s, 60°C for 30 s, and 72°C for 30 s], where data was collected during the extension at 72°C. For samples with *Canis* DNA (i.e., > 0 ng/μL), we used binary logistic regression to estimate the concentration of DNA needed to have an assumed probability that 50%, 25%, and 10% of loci would be genotyped.

### 2.2 Bioinformatics for Annotating and Error Checking Genotypes

We demultiplexed FASTQ files using Illumina's BaseSpace software with the mismatch rate between indexes set to zero. Adapter regions were trimmed using bbduk v38.94 (source: https://sourceforge.net/projects/bbmap/). We followed the read processing pipeline by Baetscher et al. (2017) and used Fast Length Adjustment of SHort reads (FLASH; Magoc and Salzberg 2011) to combine paired‐end reads. We mapped the reads using the BWA‐MEM algorithm (Li and Durbin 2009; Li 2013) to a reference FASTA file (GrayWolf_GTseq194_loci.fasta) containing the sequence of each locus for the panel (https://doi.org/10.5281/zenodo.4673716). Before calling genotypes from SAM files, we identified all SNP variants and output as a VCF file which is used to track the position of each SNP within a single locus when calling microhaplotypes. To generate the VCF file of SNP positions, we converted SAM files to sorted BAM files and used FreeBayes v0.9.21 (Garrison and Marth 2012) to discover biallelic SNP variants. We excluded variants with low‐quality scores (Q < 30) or rare minor alleles (MAF < 0.006) to avoid losing variants that would be unique to the small number of red fox samples (our taxonomic outgroup). We also excluded variants located in primer sequences.

Following variant detection, we called genotypes using the R package *microhaplot* (*Source:* https://doi.org/10.5281/zenodo.820110) using SAM files and the VCF file (WGL_GTseq_SNP_Panel_194_microhaplot.vcf) with coordinates for each SNP position per locus as input (https://doi.org/10.5281/zenodo.4673716). A genotype required a minimum read depth of 20×, and heterozygous calls required a minimum allelic ratio of 0.1. Each PCR plate included one negative PCR control to use as a second minimum read depth filter, where each genotype must exceed twice the read depth for a given locus when compared to the negative PCR control.

After we called genotypes, loci with a genotyping error rate > 5% were removed. We calculated genotyping error rates using 72 samples consisting of blood (*n* = 21), tissue (*n* = 11), and scat (*n* = 40) submitted multiple times ranging from two to seven replicates per sample. The genotyping error rate was calculated as the total number of mismatching genotypes between replicates divided by the total number of genotypes compared. We also summarized the genotyping error rate across the GTseq panel when grouped by sample type (i.e., blood, tissue, and scat).

The final panel consisted of 196 loci (27 F_ST_ markers, 167 diversity markers, and two sex markers). As a final verification of performance across different concentrations of DNA, we examined the distribution of on‐target read percentages across 194 autosomal loci (excluding male‐specific sex markers). For this analysis, we used DNA extracts from scat that were previously utilized to optimize the panel for low‐quality DNA, but we included only samples with at least one successfully genotyped locus. The distribution of on‐target read percentages across three subsets of samples with host‐specific DNA concentrations ranging from 0.00–85.37 ng/μL, 0.05–85.37 ng/μL, and 1.00–85.37 ng/μL was used to explore how disproportionate reads were distributed across loci as DNA concentrations were reduced.

### 2.3 Bioinformatics for Sex Inference

To assign sex, we used a combination of perl scripts and the final set of 194 autosomal input probe sequences for GTseq loci (GTseq_Genotyper_v3.pl., canid_genotypic‐sex_test.pl., GTseq_GenoCompile_v3.2.pl, and input_probeseqs_GTseq194.csv; https://doi.org/10.5281/zenodo.4673716). We first calculated the expected number of on‐target reads by multiplying the total number of on‐target reads for a given sample by an assumed proportion of on‐target reads for a given sex marker. For sex markers Clu_ddx3Y‐211,920 and Clu_usp9Y‐75,200, the fraction of on‐target reads attributed to males was assumed to be 0.005 and 0.003, respectively.

We then calculated the ratio between the expected number of on‐target reads and the actual number of on‐target reads for the sex marker. We assigned a sex category of “unassigned” if read depth was < 10× or when ratios were between 0.1 and 0.2. We classified the sample as male if the ratio was greater than 0.2, and ratios < 0.1 were classified as female.

We assessed the accuracy of sex assignment by comparing two sex markers to field‐based sex for 205 gray wolf samples. Field‐based sex was assigned for each wolf during collaring events, relocation efforts, or necropsy. Samples were collected from wolves on Isle Royale (*n* = 5 tissue, *n* = 27 blood, and *n* = 12 scat) and the Upper Peninsula of Michigan (*n* = 161 tissue). To confirm a sex assignment, we required that both sex markers must contain a call as male or female and that both sex markers agreed with each other.

### 2.4 Assessment of GTseq Panel Performance: Species ID

We assessed the performance of the final GTseq panel of 194 autosomal loci, which included 27 population *F*_*ST*_ markers and 167 diversity markers (microhaplotype and high MAF loci). We cross‐validated our GTseq panel's ability to assign species by using a new set of 159 blood and tissue samples collected from canids across the WGL not used during SNP discovery (see Zenodo repository for sample locations). We prepared high‐quality DNA of gray wolves (*n* = 56), eastern wolves (*n* = 36), coyotes (*n* = 46), domestic dogs (*n* = 14), and red foxes (*n* = 7) using the same library preparation steps outlined for high‐quality DNA from the optimization run of the panel. Taxonomic assignments for domestic dogs, red foxes, and gray wolves from Minnesota and Michigan were based on morphological assessment, while gray wolves, eastern wolves, and coyotes from Ontario, where interspecies breeding is more common, were all previously assigned using a microsatellite panel (Wheeldon 2009; OMNR unpublished data).

We used the R package *Adegenet* to summarize the observed and expected heterozygosity for each locus and plotted these values against each other using a best fit line assuming a linear trend grouped by taxonomy. Further, to explore if ascertainment bias was present by initially designing diversity markers using just gray wolf RADseq samples, we used the R package *ggplot2* (Wickham 2016) to visualize boxplots of observed heterozygosity grouped by taxonomy.

We assessed genetic structure using a principal component analysis (PCA) with R package *factoextra* (Kassambara and Mundt 2016) and calculated Nei's pairwise *F*_*ST*_ (Nei 1973) values with the R package *Hierfstat* (Goudet 2004). We further excluded red fox and used the Bayesian genetic clustering algorithm in *STRUCTURE* v2.3.4 (Pritchard et al. 2000) to estimate the probability of cluster assignment. We assumed one to ten clusters (*K*) with 10 iterations for each. We implemented a burnin period of 10000 and 10000 MCMC repetitions in the admixture model with all default parameters, assuming allele frequencies were correlated. To identify the most supported number of clusters, we used *STRUCTURE Harvester* (Earl and von Holdt 2012) using both the highest mean LnP(*K*) and the highest Delta *K* value.

To confirm our GTseq panel was in agreement with the original samples used for SNP discovery, we compared population structure for a subset of gray wolves (*n* = 16), eastern wolves (*n* = 6), coyotes (*n* = 20), and admixed Ontario canids (*n* = 15) previously assigned using RADseq (Heppenheimer et al. 2018) and that we resequenced using the newly developed WGL GTseq panel. The RADseq dataset was filtered for a set of neutral SNPs using the same pipeline previously described above. We retained only biallelic loci that had a MAF > 0.10, did not significantly deviate from Hardy–Weinberg equilibrium assumptions (–hwe 0.001), had a maximum of 5% missing data (–geno 0.05), were in linkage equilibrium (indep‐pairwise 50 5 0.5) and were autosomal (–chr 1‐38).

We used program *STRUCTURE* to compare probabilities of assignment when using the RADseq and GTseq datasets containing the same gray wolves, eastern wolves, coyotes, and admixed canids from Ontario. Both datasets used identical run parameters (*K* = 1–6, burnin iterations = 10000, MCMC reps = 10000) using default parameters in the admixture model and assumed allele frequencies were correlated.

#### 2.4.1 Assessment of GTseq Panel Performance: Identity

To understand the probability of two individuals having identical genotypic profiles from the GTseq SNP panel, we calculated the probability of identity (PID) assuming related individuals are present within the sample population, hereafter PID_sibs_ (Waits et al. 2001). To provide an estimate of PID_sibs_ representing a biologically relevant set of samples, we calculated PID_sibs_ from 20 gray wolves from Isle Royale National Park (IRNP), Michigan, where the ability to assign individual identification is vital for long‐term monitoring. We also randomly selected 1 to 194 loci on the GTseq panel across 1000 iterations and calculated PID_sibs_ to understand how the GTseq panel performs when varying levels of missing data are present.

It is also useful to provide comparisons of PID_sibs_ relative to other commonly implemented methods for identity analysis. Thus, we compared estimates of PID_sibs_ from 194 loci on our GTseq panel, 18 microsatellite loci, and 684 RADseq SNPs genotyped from the same 20 wolves from IRNP (see Zenodo repository for details on RADseq and microsatellites).

#### 2.4.2 Assessment of GTseq Panel Performance: Kinship

We explored the power of the GTseq SNP panel for inferring relationships using the R package *CKMRsim* (Anderson 2022), which uses Monte Carlo methods to assess the false positive rate (FPR) of assigning an incorrect relationship across the false negative rate (FNR) of not assigning a relationship when individuals are related. These rates are based on simulated genotypes using the number of loci, alleles per locus, and frequency of each allele calculated from 20 gray wolves from IRNP. We estimated FPR as the probability of falsely assigning a comparison as unrelated when the true assignment was either parent‐offspring or full siblings using 194 GTseq SNP panel loci, 18 microsatellite loci, and 684 RADseq SNP loci. We assumed a genotyping error rate of 5% for RADseq, although it can range from 5% to > 15% (Mastretta‐Yanes et al. 2014). Further, a genotyping error rate of 1% was assumed for microsatellites based on previous studies using a similar set of microsatellite loci for canids of the WGL (Rutledge et al. 2010a), while GTseq loci were assumed to be 0.39% based on our findings within this study.

To understand how FPRs are influenced by the number of loci included in the GTseq Panel, we randomly retained subsets of loci (50% or 97 loci, 40% or 78 loci, 30% or 58 loci, 25% or 49 loci, and 20% or 39 loci). From the simulated datasets generated from 20 IRNP wolves with varying loci retained, we estimated the number of false positive assignments between two individuals as unrelated when their true relationship was either parent‐offspring or full siblings.

## 3 Results

We discovered 522,782 loci from RADseq, of which we retained 50,549 *F*_*ST*_ markers and 9947 diversity markers after filtering. Two genes (ddx3Y and usp9Y) on the Y chromosome were used to select loci for sex markers.

After selection screening, we identified 49 *F*_ST_, 254 diversity, and 6 sex markers to include on the preliminary GTseq panel. After two rounds of primer optimization and removing loci with greater than a 5% error rate, the final GTseq panel retained 196 loci consisting of 27 *F*_ST_ markers, 167 diversity markers, and 2 sex markers. We identified 801 biallelic SNPs across the 194 autosomal markers. From samples genotyped in replicate, we observed a genotyping error rate of 0.39% (69 mismatches across 17,578 genotypes) for all sample types. When summarized by sample type, the genotyping error rate for blood, tissue, and scat was 0.19% (20 mismatches per 10,268 genotypes), 0.07% (3 mismatches per 4125 genotypes), and 1.44% (46 mismatches per 3185 genotypes), respectively.

The median percentage of on‐target reads per locus ranged from 0.03% to 6.88% when using samples with host‐specific DNA concentrations ranging from 0.00 to 85.37 ng/μL. When excluding DNA concentrations below 0.05 ng/μL, the difference between the maximum and minimum percentage of on‐target reads for loci was reduced (max = 3.62%, min = 0.25%; https://doi.org/10.5281/zenodo.4673716).

### 3.1 Methodological Manipulations Have Varying Effects

**FIGURE 2:**
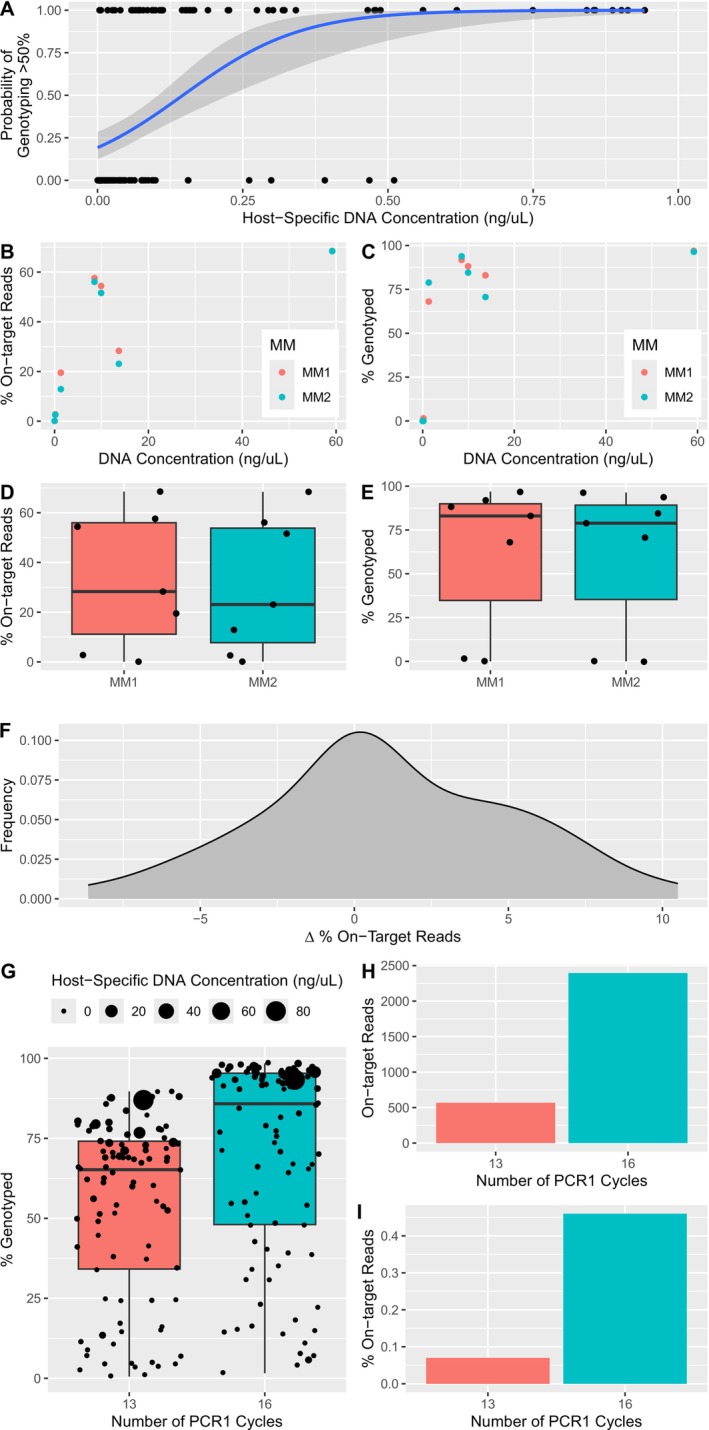
(A) Probability of genotyping greater than 50% of loci relative to host DNA concentrations (figure only presents data < 1 ng/μL to better visualize the trend, but all data were included when generating the binary logistic regression). (B) The percentage of on‐target reads relative to DNA concentration when using one (MM1) or two (MM2) primer master mixes. (C) The percentage of loci genotyped relative to DNA concentration when using MM1 or MM2. (D) Boxplot of the percentage of on‐target reads categorized by MM1 and MM2. (E) Boxplot of the percentage of loci genotyped categorized by MM1 or MM2. (F) Density plot of delta percentage of on‐target reads where negative values indicate higher on‐target reads when DNA was treated with Exonuclease I and shrimp alkaline phosphatase. (G) Boxplot of the percentage of loci genotyped when using 13 or 16 PCR1 cycles and point size reflects host‐specific DNA concentration. Number of on‐target reads (H) and percentage of on‐target reads (I) for the negative PCR control when using 13 or 16 PCR1 cycles.

We found no significant difference between the percentage of on‐target reads with and without ExoSAP treatment of genomic DNA (*t*_45_ = 1.776, *p* = 0.08) or the overall quality of library sequencing (Figure 2, Table 1). Splitting PCR1 into two reactions, each containing half the primer pairs of the GTseq panel, did not influence the percentage of on‐target reads (*t*_12_ = 0.159, *p* = 0.88; Figure 2). We did find that increasing the number of PCR1 cycles from 13 to 16 increased the percentage of loci genotyped (*p* < 0.01) as well as the number and percentage of on‐target reads for the negative PCR control (Figure 2).

Of the 556 scat samples extracted, 31.0% (76.0% in winter months, 22.0% in summer months) had a DNA concentration > 0 ng/μL estimated from qPCR and were used to estimate the concentration of DNA needed to have an assumed probability that 50% (Figure 2), 25%, and 10% of loci would be genotyped (https://doi.org/10.5281/zenodo.4673716). All three binary logistic regressions had a significant positive trend, whereby as DNA concentration increased, so did the probability of genotyped loci. Specifically, to have a 95% probability that we would genotype at least 50%, 25%, and 10% of loci, this required a minimum of 0.73 ng/μL, 0.45 ng/μL, and 0.14 ng/μL of DNA, respectively.

### 3.2 WGL GTseq Panel Can Differentiate Unique Canid Lineages

**FIGURE 3:**
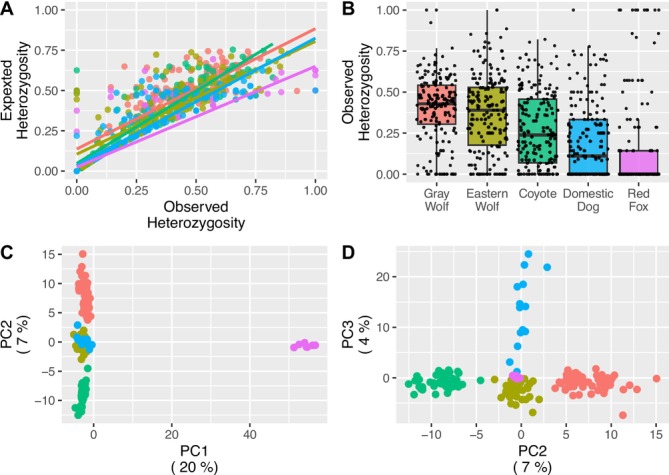
(A) Observed and expected heterozygosities with assumed linear best fit lines colored by taxonomic group (red = gray wolf, yellow = eastern wolf, green = coyote, blue = dog, pink = red fox). (B) Boxplot of observed heterozygosity for each taxonomic group. Principal component analysis of gray wolves, eastern wolves, coyotes, domestic dogs, and red foxes for principal components 1 and 2 (C) and principal components 2 and 3 (D), with variance explained for each principal component shown in parentheses.

Of the 194 autosomal markers, we observed a linear positive trend between observed and expected heterozygosity (Figure 3). In addition, gray wolves carried the highest observed heterozygosity and decreased as canids were more taxonomically distant from gray wolves, with the exception of domestic dogs (Figure 3).

**FIGURE 4:**
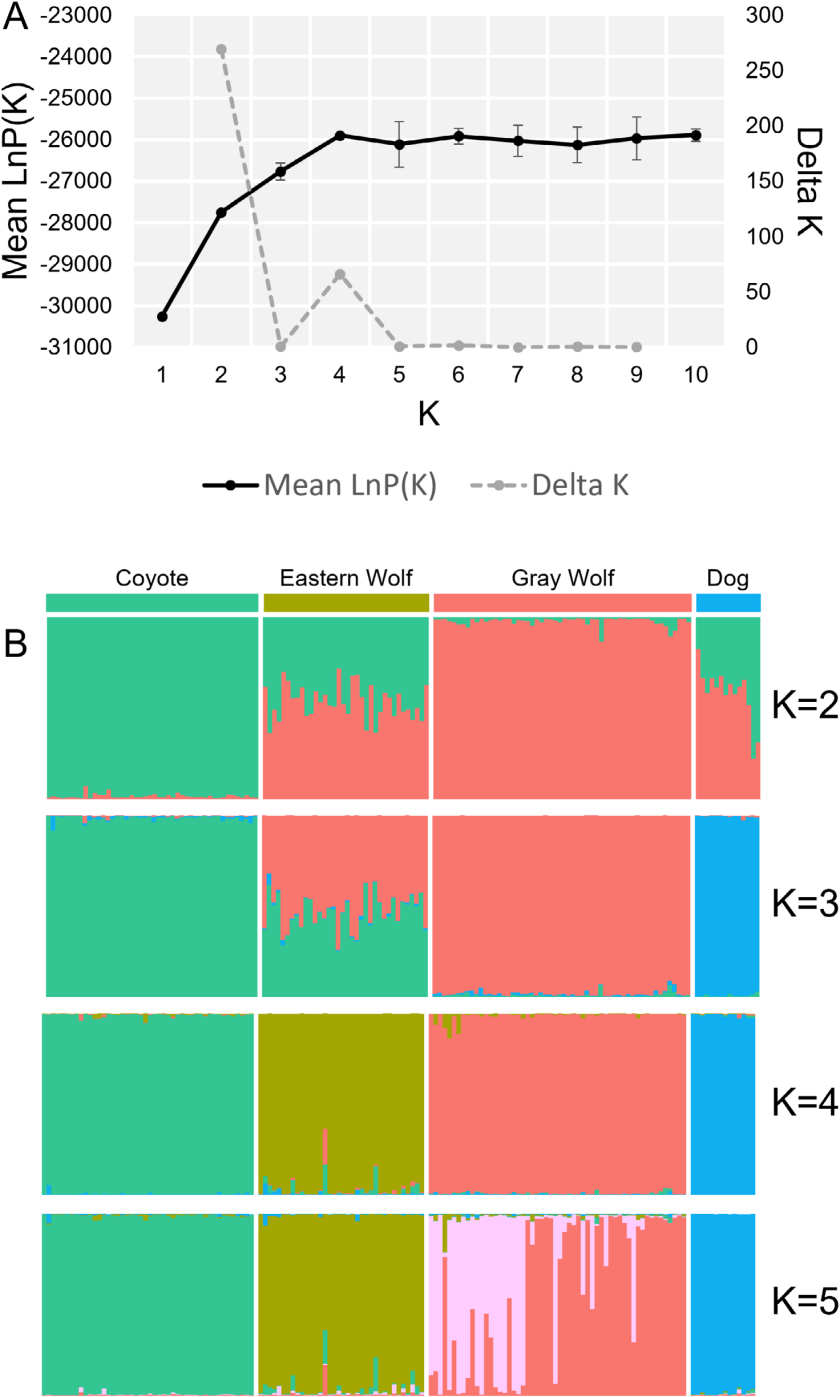
(A) Mean LnP(K) and delta K from a STRUCTURE analysis assuming one through 10 clusters (K) each with 10 replicates. (B) Structure plot organized by taxonomic grouping where each color represents a unique cluster and the height of the colored bars represents the probability of assignment to the cluster.

Qualitatively, a PCA of all canids differentiated red fox from all other canids on PC 1 (20% of the variance), while PC 2 (7% of variance) separated gray wolves from coyotes, with eastern wolves as an intermediate between both groups, and dogs clustered uniquely on PC 3 (4% of variance; Figure 3). Pairwise *F*_*ST*_ values were also in agreement with the PCA, where the highest differentiation was observed between red fox and all other canids, while eastern wolves were an intermediate between coyotes and gray wolves (https://doi.org/10.5281/zenodo.4673716). The Bayesian probabilities of assignment were concordant with the PCA where all canids had high assignments to their respective clusters (Figure 4).

**FIGURE 5:**
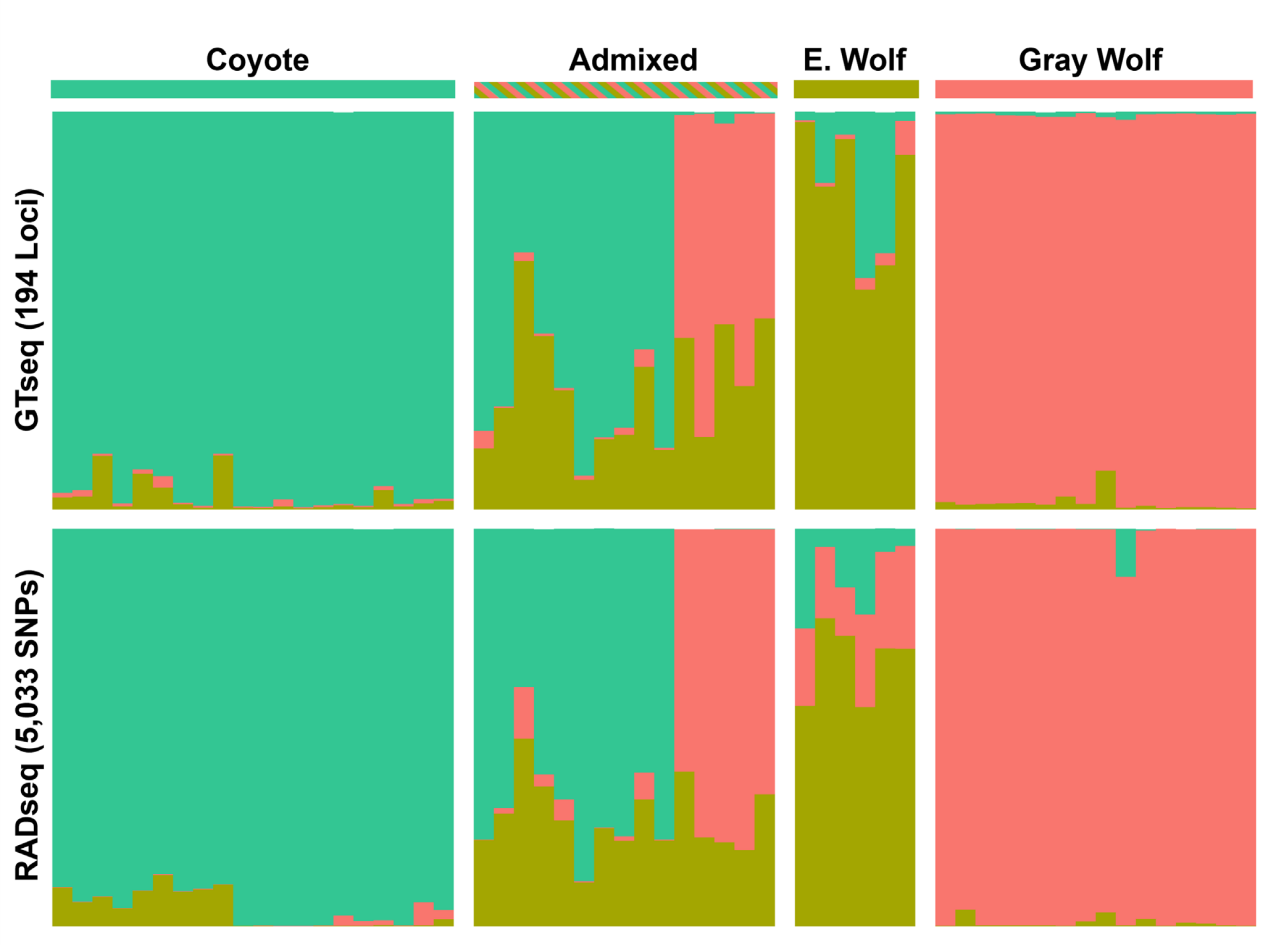
Barplots comparing the probability of assignments using 194 loci from GTseq to 5033 SNPs from RADseq calculated from program STRUCTURE when assuming three clusters.

When comparing Bayesian cluster assignments between GTseq and RADseq, we found high concordance across both methods (Figure 5). Although eastern wolves always carried the majority of cluster assignments to representative eastern wolves, in both methods, we found greater variation relative to other canids (i.e., gray wolves, coyotes, and admixed) across methods.

### 3.3 WGL GTseq Panel Can Infer Sex

Of the 205 canid samples genotyped with sex assignments from direct field observations, 25 were unassigned due to either conflicting sex assignments at the two sex markers (*n* = 5) or one or both sex markers were not genotyped (*n* = 20). Of the 180 canids with an assigned sex from sex markers, 175 matched their field assigned sex (97.2%) and five did not (2.8%). Across sample types, the proportion of uncalled samples was highest for scat, followed by tissue, then blood (https://doi.org/10.5281/zenodo.4673716). Overall, 175 of the 205 canid samples were successfully inferred with the correct sex (85%).

### 3.4 WGL GTseq Panel Can Identify Unique Individuals

**FIGURE 6:**
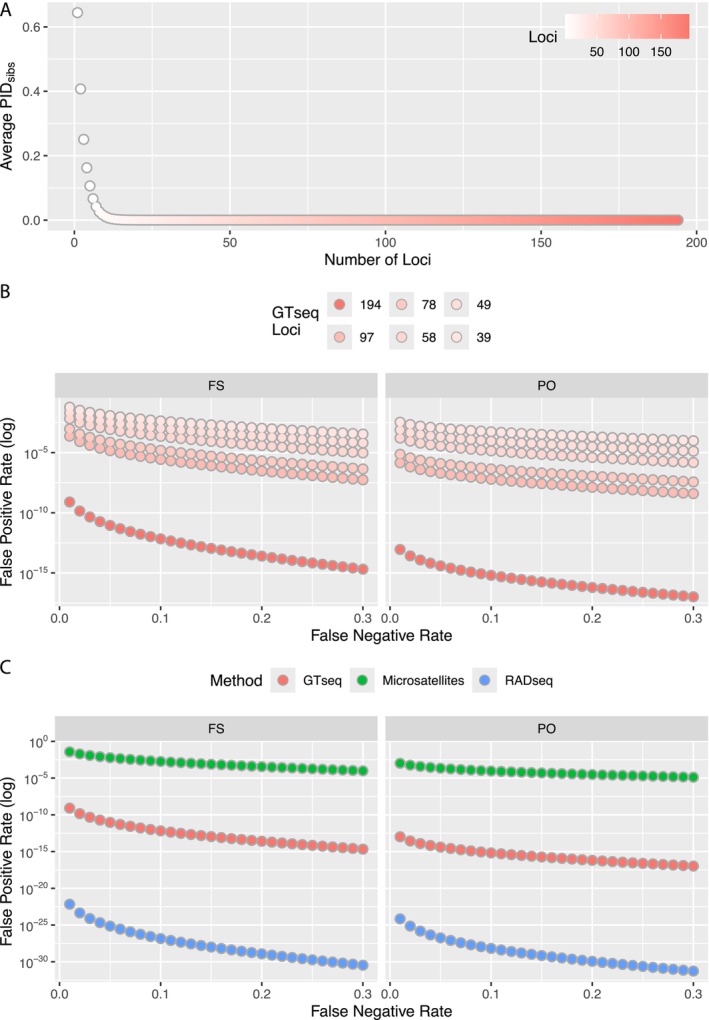
(A) Average PID_sibs_ when randomly retaining 1 to 194 loci across 1000 iterations. (B) Simulated false positive rates across false negative rates for GTseq microhaplotypes when randomly retaining a set number of loci when assigning full‐siblings (FS) and parent‐offspring (PO) relationships. (C) Simulated false positive rates across false negative rates when using 194 GTseq microhaplotypes (red), 18 microsatellite loci (green), and 684 RADseq SNPs (blue) when assigning full‐siblings (FS) and parent‐offspring (PO) relationships.

The ability for the GTseq panel to identify unique individuals required a minimum of 11 loci for the average estimate of PID_sibs_ to cross the threshold of 0.01 (Figure 6). After filtering RADseq data, we used 684 biallelic SNPs to compare against 194 autosomal markers of the GTseq panel and 18 microsatellite loci. We found that PID_sibs_ was lowest with RADseq data (2.37 × 10^−155^), GTseq was intermediate (6.71 × 10^−41^), and microsatellites resulted in the highest PID_sibs_ value (1.02 × 10^−7^).

### 3.5 WGL GTseq Panel Can Assign Relationships

Across all false negative rate values, we report that the false positive rate was highest in microsatellites, followed by GTseq, with the RADseq dataset the least likely to contain false positives (Figure 6). Specifically, when we assumed a false negative rate of 0.01, the false positive rate of assigning two individuals as unrelated when their true assignment was parent‐offspring was 7.55 × 10^−25^ for 684 RADseq loci, 9.34 × 10^−14^ for 194 GTseq loci, and 1.06 × 10^−3^ for 18 microsatellite loci.

We also found the GTseq panel maintained very low false positive rates with small subsets of loci (Figure 6). For example, assuming a false negative rate of 0.01, the false positive rate of assigning two individuals as unrelated when their true relationship is parent‐offspring is approximately 3.33 × 10^−4^ when using 58 GTseq loci (30%). However, by increasing the number of loci to 97 (50%) and assuming the same false negative rate, the false positive rate decreases to 9.77 × 10^−7^.

## 4 Discussion

Improving the applicability and quality of GTseq panel development requires transparency about common optimization challenges. In this discussion, we present various strategies and assays to enhance sequencing success. GTseq has great potential in conservation due to its ability to process thousands of samples efficiently (Bootsma et al. 2020), cost‐effectiveness (Campbell et al. 2015; Meek and Larson 2019), and capacity to produce consistent, repeatable data. GTseq has been applied in various studies using both high‐quality DNA (Campbell et al. 2015; May et al. 2020; Li et al. 2023) and low‐quality DNA (Eriksson et al. 2020; Schmidt et al. 2020; Burgess et al. 2022; Hayward et al. 2022; Arpin et al. 2024; Garrett et al. 2024). These applications highlight its versatility, particularly for noninvasive studies. Here, we outline strategies to improve the success of GTseq panels for noninvasively collected samples, focusing on methodological recommendations and concerns.

### 4.1 Methodological Considerations

Overall, the concentration of DNA was the most important consideration for sequencing quality and the percentage of loci genotyped, where both improved as DNA concentration increased. A previous noninvasive study found DNA concentration did not influence the percentage of loci genotyped, but their average DNA concentrations exceeded 10 ng/μL for all sample types (Schmidt et al. 2020). For our study, host‐specific DNA concentrations estimated from scat had a wide range of concentrations (0.00–85.37 ng/μL). From this range, we have found a strong positive relationship between DNA concentration and the percentage of loci genotyped. Further, binary logistic regression is a useful statistical tool that allows us to determine the probability of genotyping a predefined percentage of loci based on the estimated host‐specific DNA concentration. We can use these probabilities to set thresholds for removing samples with DNA concentrations that will likely return unusable data, or an insufficient number of genotypes for a specific question, thus saving time and money.

Quantifying host DNA concentrations from qPCR is also necessary to remove samples that will return little to no usable sequencing data, especially if future runs solely contain samples from low‐quality sources. This was evident from a single library we submitted, which contained all low‐quality samples without a qPCR filter. The sequencing run nearly failed due to a high proportion of off‐target reads and poor quality, resulting in very little genotyping data; therefore, it was not included in this study. Illumina sequencing platforms are sensitive to the diversity and the predefined length of fragments that are sequenced, and if most samples contain large concentrations of primer dimers relative to on‐target reads, this may lead to the sequencing run failing. Primer dimers are shorter in length compared to on‐target reads. As a result, these shorter reads are sequenced until the template is exhausted, preventing further base incorporation and ultimately reducing the overall quality of the sequencing run. Further, if a subset of primer dimers amplifies more efficiently, these sequences will be overrepresented on the flow cell and reduce cluster diversity, making it difficult to differentiate clusters and lowering the quality of the run.

We also increased the number of cycles for PCR1 from 13 to 16 and found that the average number of loci genotyped increased, with the caveat that so did the number of on‐target reads detected within our negative PCR control. Because we used combinatorial dual indexing, we cannot rule out if the source of increased on‐target reads was due to well‐to‐well contamination, tag‐jumping, or a combination of both. If tag‐jumping, rather than contamination, is the main source of on‐target reads found within negative PCR controls, this issue could be mitigated by using unique dual indexing for future sequencing runs. Regardless of the source, this result highlights the importance of accounting for incorrectly assigned reads through the use of negative PCR controls. We used a read depth of 20 as the minimum threshold to call a genotype but also included a secondary threshold of double the read depth of on‐target reads observed per locus for negative PCR controls. PCR1 cycles have been increased up to 35 cycles for GTseq panels (Burgess et al. 2022) and with genotyping error rates under 1%. However, we found that increasing our PCR1 cycle by three resulted in on‐target reads being over four times higher for our negative PCR control, and without accounting for this, it may lead to higher genotyping error. We consider approaching this manipulation with caution and emphasize the importance of including and reporting negative PCR controls to detect and account for incorrectly assigned reads.

We also gained several insights into factors that did not improve the performance of the GTseq panel. For instance, consistent with Bootsma et al. (2020), we found that treating DNA with ExoSAP did not increase the percentage of on‐target reads. Similarly, splitting our primer master mixes had no effect on either the percentage of on‐target reads or the percentage of loci genotyped. However, because primers were randomly assigned to primer mixes in our approach, future work could investigate whether a strategic method might improve the number of on‐target reads during sequencing, such as identifying specific primer interactions to exclude from the same primer master mix.

### 4.2 GTseq Panel Performance

Canids of the western Great Lakes have experienced historic and contemporary interspecific mating events that have led to many admixed individuals across the landscape. Specifically, eastern wolves near and around Algonquin Provincial Park, Ontario can mate with coyotes and gray wolves, thereby acting as a bridge of genetic exchange between all three taxonomic groups (Rutledge et al. 2010b). Due to the complex level of admixture across this landscape, it was critical our panel could differentiate species groups. We have demonstrated the GTseq panel has the power to differentiate both at a species/subspecies scale (gray wolf, eastern wolf, coyote, domestic dog, and red fox) and identify substructure within gray wolves (Figure 4). Further, when the same samples were compared between RADseq and GTseq, probabilities of assignment were very similar, including eastern wolves which were not assigned with high probability to any cluster, but consistently so when using both RADseq and GTseq. Although outside the scope of this study, to ensure consistency in species identification, future work should incorporate all current molecular techniques used to identify canids across the WGL (i.e., microsatellites, GTseq, RADseq, whole‐genome sequencing) as well as understanding how different compositions of reference samples may bias species assignments for unknown canids needing a species identification (Shringarpure and Xing 2014).

To evaluate the GTseq panel's utility for other canids, we estimated heterozygosity across gray wolves, eastern wolves, coyotes, domestic dogs, and red foxes. Heterozygosity declined with increasing taxonomic dissimilarity to gray wolves (Figure 3), underscoring ascertainment bias arising from the selection of highly variable markers (i.e., high allele counts and minor allele frequencies) within gray wolves. Interestingly, domestic dogs exhibited lower observed heterozygosity than expected based solely on taxonomic dissimilarity, which may reflect their inherently lower heterozygosity compared to gray wolves (Marsden et al. 2015).

Ascertainment bias is a common consequence of designing genetic markers for specific taxa or research questions, leading to reduced diversity when applied to non‐source species (Selkoe and Toonen 2006). For instance, using microsatellites on non‐source species often results in a loss of allelic diversity (Primmer et al. 1996; Ellegren et al. 1997; Neff and Gross 2001; Wright et al. 2004). This is particularly relevant in canids, where markers originally designed for domestic dogs have been applied to gray wolves (Hervey et al. 2021), eastern wolves (Grewal et al. 2004), coyotes (Otis et al. 2017), and red foxes (Swanson et al. 2005).

Similarly, SNP‐based panels like GTseq are tailored to specific taxa or questions, and their application beyond the intended application can introduce biases. For example, Bootsma et al. (2020) reported slightly lower estimates of genetic diversity in walleye populations that constituted a smaller proportion of the reference population used to design the GTseq panel. Consistent with this, our findings highlight ascertainment bias in genetic diversity across taxa. We emphasize that interspecific comparisons of genetic diversity should be avoided when using panels not specifically designed for such purposes.

When selecting markers for individual identification and parentage assignment, there are many avenues of filtering parameters to use, such as high minor allele frequency (May et al. 2020) and heterozygosity (Baetscher et al. 2017) or a combination of these filters (Bootsma et al. 2020). Both have been shown to provide loci capable of reidentifying individuals and their relationships among each other, but we wanted to select microhaplotype loci that would be more resilient to loci becoming fixed relative to biallelic loci. This is especially important for populations such as the wolves of IRNP, where inbreeding will likely increase through time (Hervey et al. 2021).

The extremely low PID_sibs_ value estimated for gray wolves on IRNP indicates the GTseq panel will have a very high power to differentiate unique individuals. The panel also contains the necessary power to assign relationships for gray wolves given the low false positive rates (FPR) observed for the 194 autosomal markers, which were lower than the FPR when using 18 microsatellite loci. Importantly, even when only 30% of the GTseq panel genotyped, we were able to maintain an FPR comparable to 18 microsatellite loci.

To help inform pedigree reconstruction, the final GTseq panel contained two sex markers after all optimization runs. These markers demonstrated high accuracy (97.2%) when amplification was successful, but there were instances where field‐based sex assignments differed from genetic assignments. Discrepancies between field and genetically inferred sex could be due to minor levels of contamination, low DNA concentrations, or a combination of those factors. An alternative explanation could also be erroneous field‐based assignments of sex. For example, we know of one wolf initially identified as male during a GPS collaring event that was later genetically identified as female. Multiple reidentifications of this wolf from independent scat samples provided further confidence in the genetic assignment of female.

For sex inference, we adopted an approach that amplified male‐specific markers located on the Y‐chromosome. This method leverages large deviations in the distribution of on‐target reads between males and females to infer the sex at each marker. We used strict criteria to infer sex, requiring both markers to be genotyped and in agreement. However, 20 out of 205 (9.8%) samples failed to infer sex at one of the two sex markers. Had we included a greater number of sex markers, we may have been able to relax our sex‐typing criteria and infer sex for a larger percentage of samples.

Alternative methods for sex inference may address these limitations. For example, in sitka black‐tailed deer ( *Odocoileus hemionus sitkensis* ), markers with sex‐biased allele frequencies were selected and included on their GTseq panel (Burgess et al. 2022). Then sex was inferred using a Bayesian clustering approach using program *STRUCTURE*. In addition to using markers directly on a GTseq panel to infer sex, there are alternative approaches like the use of qPCR. For instance, in otters ( *Lutra lutra* ), a qPCR assay has been developed using the ZFX region shared between males and females as a positive control while also amplifying the male‐specific ZFY region where the presence indicates a male (O'Neill et al. 2013).

Including sex‐typing markers on our GTseq panel proved to be a convenient and cost‐effective strategy by eliminating the need for an additional qPCR assay. This approach is particularly advantageous when scaling up to process thousands of samples. However, its reliance on strict genotyping criteria led to unknown sex inference for some samples. In comparison, Bayesian clustering of allele‐frequency‐based sex markers provides robustness by incorporating multiple loci and probability‐based assignments.

## 5 Conclusion

The western Great Lakes GTseq panel was capable of genotyping samples from a wide range of DNA concentrations and of varying quality (i.e., scat, blood, and tissue). Most notably, we found qPCR to be a helpful method to remove samples with no amplifiable host DNA. Noninvasive studies should use qPCR to avoid sequencing runs being diluted by poor‐quality samples, resulting in unusable data. Also, increasing the number of PCR1 cycles increased the average percentage of loci genotyped, with the caveat that so did incorrectly assigned reads, as detected within negative PCR controls. We caution against the use of increasing PCR cycle number unless accounted for through the use of negative PCR controls.

Further, the panel was found to have high accuracy when differentiating gray wolves from other canids commonly found in the western Great Lakes and with similar accuracy to a much higher resolution dataset generated from RADseq. The GTseq panel also provided a lower PID_sibs_ and false positive rate for identity analysis and kinship, respectively, relative to 18 microsatellite loci with the added benefit that the GTseq genotypes were called in a consistent fashion (0.39% error rate) without bias due to an automated microhaplotype calling pipeline. Our hope is that this GTseq panel will promote collaboration among laboratories using the same GTseq panel and benefit long‐term studies by generating genetic data in a consistent fashion.

## Author Contributions

**Samuel D. Hervey:** conceptualization (equal), data curation (equal), formal analysis (lead), funding acquisition (supporting), investigation (equal), methodology (equal), project administration (equal), validation (equal), visualization (equal), writing – original draft (lead), writing – review and editing (lead). **Bridgett M. vonHoldt:** conceptualization (equal), data curation (equal), formal analysis (supporting), investigation (supporting), methodology (supporting), project administration (supporting), writing – original draft (equal), writing – review and editing (equal). **Mark C. Romanski:** conceptualization (supporting), data curation (equal), funding acquisition (equal), project administration (equal), writing – original draft (equal), writing – review and editing (equal). **Tyler J. Wheeldon:** data curation (equal), writing – original draft (equal), writing – review and editing (equal). **Brent R. Patterson:** data curation (equal), writing – original draft (equal), writing – review and editing (equal). **Kristin E. Brzeski:** conceptualization (equal), data curation (equal), formal analysis (supporting), funding acquisition (lead), investigation (equal), methodology (supporting), project administration (lead), supervision (lead), writing – original draft (equal), writing – review and editing (equal).

## Conflicts of Interest

The authors declare no conflicts of interest.

## Data Availability

Supporting Information and files required for GTseq genotyping along with GTseq, RADseq, and microsatellite data needed to replicate the analysis were deposited into Zenodo DOI: https://doi.org/10.5281/zenodo.4673716. Wolf tissue samples from Michigan's Upper Peninsula were provided under a data‐sharing agreement with the Michigan Department of Natural Resources.
